# Western diet induces a shift in microbiota composition enhancing susceptibility to Adherent-Invasive *E. coli* infection and intestinal inflammation.

**DOI:** 10.1038/srep19032

**Published:** 2016-01-08

**Authors:** Allison Agus, Jérémy Denizot, Jonathan Thévenot, Margarita Martinez-Medina, Sébastien Massier, Pierre Sauvanet, Annick Bernalier-Donadille, Sylvain Denis, Paul Hofman, Richard Bonnet, Elisabeth Billard, Nicolas Barnich

**Affiliations:** 1 Clermont Université, M2iSH, UMR 1071 INSERM/Université d’Auvergne, Unité Sous Contrat 2018 Institut National de la Recherche Agronomique, Clermont-Ferrand, France; 2 Clermont Université, Université d’Auvergne, Centre de Recherche en Nutrition Humaine Auvergne, EA 4678 CIDAM, Conception Ingénierie et Développement de l’Aliment et du Médicament, Clermont-Ferrand, France; 3 Digestive Surgery Department, Centre Hospitalier Universitaire, Clermont-Ferrand 63000, France; 4 UR454 Microbiology Division, INRA, Research Centre of Clermont-Ferrand-Theix, 63122 Saint Genès-Champanelle, France; 5 Laboratory of Clinical and Experimental Pathology and Hospital-Related Biobank (BB 0033-00025), Pasteur Hospital, and IRCAN CNRS UMR 7284, Inserm U1081, Nice Sophia Antipolis University, France; 6 Bacteriology Department, Centre Hospitalier Universitaire, Clermont-Ferrand 63000, France; 7 Institut Universitaire de Technologie, Génie Biologique, Aubière, France

## Abstract

Recent advances have shown that the abnormal inflammatory response observed in CD involves an interplay among intestinal microbiota, host genetics and environmental factors. The escalating consumption of fat and sugar in Western countries parallels an increased incidence of CD during the latter 20^th^ century. The impact of a HF/HS diet in mice was evaluated for the gut micro-inflammation, intestinal microbiota composition, function and selection of an *E. coli* population. The HF/HS diet created a specific inflammatory environment in the gut, correlated with intestinal mucosa dysbiosis characterized by an overgrowth of pro-inflammatory Proteobacteria such as *E. coli*, a decrease in protective bacteria, and a significantly decreased of SCFA concentrations. The expression of GPR43, a SCFA receptor was reduced in mice treated with a HF/HS diet and reduced in CD patients compared with controls. Interestingly, mice treated with an agonist of GPR43 were protected against DSS-induced colitis. Finally, the transplantation of feces from HF/HS treated mice to GF mice increased susceptibility to AIEC infection. Together, our results demonstrate that a Western diet could aggravate the inflammatory process and that the activation of the GPR43 receptor pathway could be used as a new strategy to treat CD patients.

Evidence has demonstrated that in inflammatory bowel diseases (IBD), including Crohn’s disease (CD) and ulcerative colitis (UC), dysfunction of the immune response to gut microbiota occurs in context of host genetic predisposition. CD is a chronic and commonly disabling inflammatory disorder of the intestine, and its prevalence and incidence are increased in developed countries^1^. Because etiology of this disease remains poorly understood, no specific treatment is available. CD preferentially affects young adults and is a major public health problem because of its chronic and recurrent nature and growing prevalence.

Many investigations have focused on defining which factors associated with western lifestyle may explain dramatically increasing prevalence of new diseases such as CD in 20^th^ century^2^. Interestingly, several lines of evidence show alterations in composition of gut microbiota in patients suffering from CD. Although a specific pattern of dysbiosis in CD patients is difficult to establish, many studies have reported an increase in the abundance of Proteobacteria and Bacteroidetes and a decrease in Firmicutes^3^. A specific pathogenic group of *E. coli*, called adherent-invasive *E. coli* (AIEC), has been extensively implicated in human CD and is currently one of the most exciting players in the pathogen story^4^,^5^,^6^,^7^,^8^,^9^,^10^,^11^,^12^. Moreover, AIEC express type 1 pili that can bind to host adhesion receptor carcinoembryonic antigen-related cell adhesion molecule 6 (CEACAM6). CEACAM6 has been shown to be overexpressed in ileal CD tissue compared with healthy controls, is increased after IFN-γ or TNF-α stimulation and is upregulated by AIEC themselves^13^. Etiology of CD could be partially explained by the current genetic risk map^14^. In transgenic CEABAC10 mice expressing human CEACAMs to mimic high expression of CEACAM6 reported in CD patients, AIEC reference strain LF82 colonizes and induces strong gut inflammation in a type 1 pili dependent manner^15^.

Among factors associated with a Western lifestyle, changes in dietary habits should be investigated because escalating consumption of fat and sugar in Western countries parallels increased incidence of CD^16^,^17^. Western diet is enriched in total fat, animal proteins, n-6 polyunsaturated fatty acids and refined sugars, and it is considered as a predominant trigger implicated in development of IBD^16^. We recently demonstrated that a combination of High-Fat/High-Sugar diet (HF/HS) led to dysbiosis with increased *Bacteroides* spp and *Ruminococcus torques* in mice^18^. Consumption of “Western-style” diets that are generally low in fiber and rich in fat and digestible sugars can lead to an altered gut microbiota composition that could influence relative amounts of major metabolites produced by bacteria in the gut, such as short-chain fatty acids (SCFA). Considering widespread implications of dietary factors in development of CD, in this work, we aimed to better understand mechanisms involved in modulation of host physiology in response to “Western-style” diet, particularly the impact of a HF/HS diet in mice on (i) gut inflammation and (ii) modification of microbiota composition and function.

## Results

### Western diet causes an inflammatory environment in the digestive tract associated with microbiome perturbations

**Figure 1:**
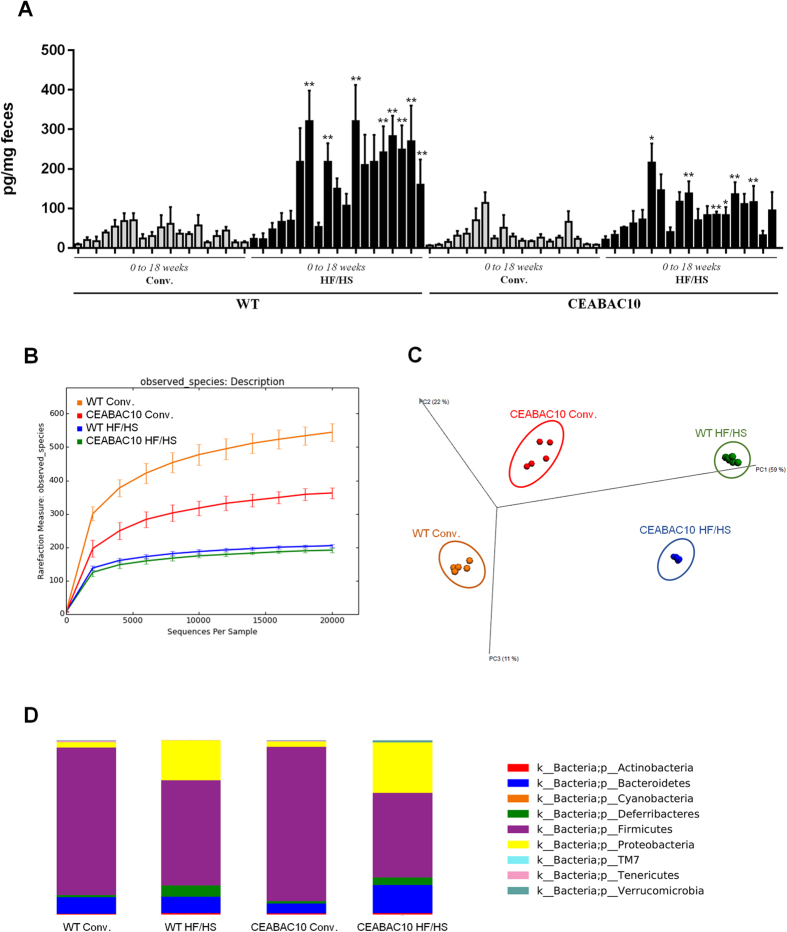
Western diet causes an inflammatory environment in the digestive tract associated with microbiome perturbations. WT and CEABAC10 mice were treated with a conventional or an HF/HS diet for 18 weeks (N = 5 per Conv. groups and N = 6 per HF/HS groups) (**A**) The fecal Lcn-2 levels were measured each week during the treatment (*P < 0.05; **P < 0.01 in WT and CEABAC10 mice under conventional and HF/HS diet; The statistically different Lcn-2 values was determined using a Mann-Whitney test between the diets, for each genotypes. (**B**) Rarefaction curves of the bacterial species richness, mean values are indicated for each group of mice. (**C**) PCoA plots based on of the unweighted UniFrac distance matrices showing clustering of mice regarding their global microbiota composition. (**D**) Relative abundance of phyla in fecal samples between groups of mice.

To investigate impact of nutrition on gut inflammation, we measured fecal Lcn-2 levels in mice that were fed a conventional (N = 5) and a High-Fat/High-Sugar (HF/HS) (N = 6) diets. The HF/HS diet, given over a period of 18 weeks, led to increased fecal Lcn-2 levels from 5 weeks until 18 weeks of treatment in both WT and CEABAC10 mice compared with mice that were fed a conventional diet, the latter of which retained a low Lcn-2 level throughout the course of the treatment. These observations showed that a Western diet creates a specific inflammatory environment in the gut, thereby increasing host susceptibility to chronic inflammatory bowel disease (Fig. 1A).

To investigate whether this micro-inflammatory environment could be due to microbiota alterations, colonic microbiota composition was compared in WT and CEABAC10 C57BL/6 mice that were fed a conventional (N = 5) or HF/HS diet (N = 6) using 16S rRNA gene sequencing. Rarefaction curves for each samples indicated that total bacteria diversity was well represented and this analysis revealed that mice fed with HF/HS diet in both genotype (WT and CEABAC10) had decreased of diversity and species richness (Fig. 1B). These observations were supported by phylogenetic distance metrics (PD_whole_tree) analyses and Chao1 richness estimator (Figure S1). A Principal Coordinate Analysis (PCoA) based on unweighted UniFrac distance matrices revealed useful information about the phylogenetic relationship and composition of colonic bacterial microbiota in the different animal groups. Samples from the same group of mice clustered together and separately from samples of the other groups of mice in the plot, with principal component scores that accounted for 59% (PC1) and 22% (PC2) of the total variance. ANOSIM test with permutations confirmed significant separation of groups, which indicated that there were clear differences in microbial composition based on diets and genotypes (Fig. 1C). Moreover, significant variations in composition of colonic microbiota were observed among WT and CEABAC10 mice treated with a conventional diet and WT and CEABAC10 mice treated with a HF/HS diet at different taxonomic levels. At the phylum level, majority of OTUs belonged to *Firmicutes* (70.6%) and *Proteobacteria* (14.5%). *Bacteroidetes* was the third most abundant (10.3%). The remainder of bacterial population had a relative abundance of < 6% in at least 4 samples. Significant predominance of *Firmicutes* was observed in WT and CEABAC10 under conventional diet (84.7% in WT Conv., 88.6% in CEABAC10 Conv., 60.4% in WT HF/HS, and 48.5% in CEABAC10 HF/HS), whereas *Proteobacteria* were more abundant in WT and CEABAC10 mice fed an HF/HS diet (3.0% in WT Conv., 3.2% in CEABAC10 Conv., 22.9% in WT HF/HS and 29.0% in CEABAC10 HF/HS). *Bacteroidetes* were particularly predominant in CEABAC10 mice receiving an HF/HS diet (9.5% WT Conv. *versus* 9.5% WT HF/HS *versus* 5.7% CEABAC10 Conv. *versus* 16.3% CEABAC10 HF/HS) (Fig. 1D). These results suggest that diet is able to shape the murine gut microbiota towards a colitogenic profile.

### Western diet favors the emergence of *E. coli* associated with the ileal, cecal and colonic mucosa

**Figure 2:**
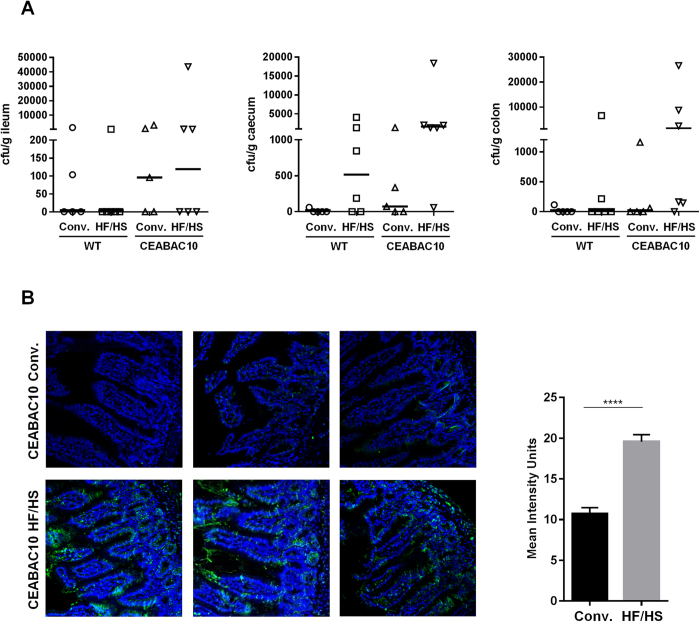
Western diet favors emergence of *E. coli* associated with ileal, cecal and colonic mucosa. (**A**) *E. coli* bacteria were isolated from the mucosa by plating on Drigalski agar plates and the number of bacteria was determined by counting the CFU. (**B**) *E. coli* colonization of the ileal mucosa of CEABAC10 mice under a conventional or an HF/HS diet was visualized by confocal microscopy and quantification was performed by assessing global fluorescence intensities using the ImageJ software. ****P < 0.0001.

Amounts of mucosa-associated *E. coli* bacteria were quantified using a culture-dependent method to analyze impact of HF/HS diet on mucosa-associated bacteria. An abnormal proportion of *E. coli* bacteria was recovered from colonic, cecal and ileal mucosa of CEABAC10 mice under a HF/HS diet and compared to mice (WT and CEABAC10) fed a conventional diet or WT mice under HF/HS diet (Fig. 2A). Moreover, higher ileal mucosa colonization in CEABAC10 mice under HF/HS diet was observed by confocal microscopy (Fig. 2B). Interestingly, CEABAC10 mice under HF/HS diet had a strong increase in mucosa-associated *E. coli* (*P* < 0.0001), showing that diet and genetic factor have a cumulative effect on mucosa-associated *E. coli* bacteria leading to dysbiosis.

### Western diet selects a colitogenic microbiota favoring AIEC colonization

**Figure 3:**
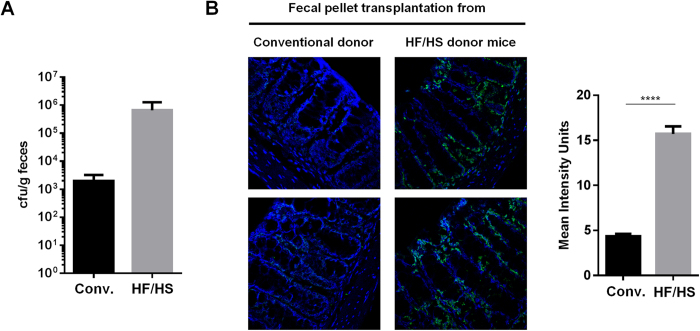
Western diet selects a colitogenic microbiota favoring AIEC colonization. (**A**) GF mice receiving a conventional or a HF/HS fecal transplantation were inoculated intragastrically with AIEC LF82. (**A**) Fecal counts of AIEC LF82 bacteria were performed (**B**) *E. coli* colonization of the colonic mucosa was compared between mice transplanted with a fecal pellet from conventional or HF/HS donor mice using confocal microscopy and quantification was performed using the ImageJ software. ****P < 0.0001.

We tested hypothesis that Germ-Free (GF) mice transplanted with fecal pellets of HF/HS donor mice would be more efficiently colonized by an AIEC strain than those receiving fecal pellets from conventional donor mice. In the fecal transplant from mice under HF/HS or conventional diets, no AIEC bacteria were detected. Before transplant, we isolated *E. coli* bacteria by plating stool on Drigalski gelosis. However, in these conditions, phenotypical analysis did not reveal any AIEC-associated adherent and invasive properties. Following intragastric inoculation, fecal counts of AIEC LF82 were higher in GF mice having received a HF/HS fecal transplantation than in GF mice transplanted with conventional microbiota (Fig. 3A). Moreover, *E. coli* colonization following AIEC LF82 inoculation, was clearly favored in mice transplanted with HF/HS fecal microbiota as shown by confocal microscopy after immunostaining (Fig. 3B). A significant 3.7-fold increase in *E. coli* immunostaining intensity signal was measured in mice transplanted with an HF/HS fecal microbiota compared to GF mice transplanted with a conventional microbiota (*P* < 0.0001). However, no significant differences in IL-6, KC, TNF-α and IL-1β cytokine release were measured between mice transplanted with HF/HS stool or conventional stool following AIEC colonization (data not shown). Together, these results suggest that dysbiotic microbiota obtained after the consumption of a HF/HS diet is more susceptible to be colonized by AIEC.

### Western diet increases susceptibility to chemically-induced colitis

**Figure 4:**
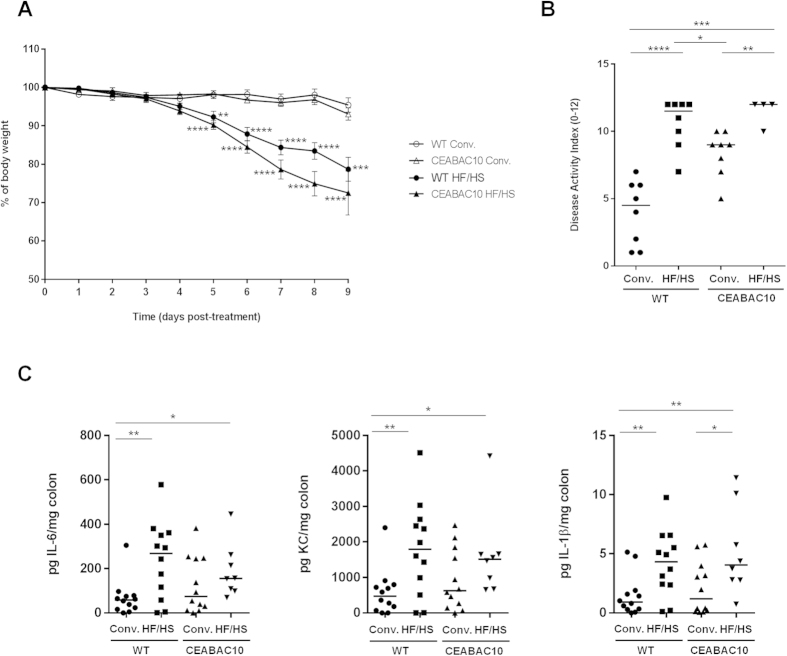
Western diet increases susceptibility to chemically-induced colitis. (**A**) Evolution of body weight, (**B**) DAI at day 9 after DSS treatment and (**C**) IL-6, KC and IL-1β secretion by colonic mucosa. *P < 0.05; **P < 0.01; ***P < 0.001, ****P < 0.0001 (compared to WT mice under a conventional diet).

We tested hypothesis that mice receiving a HF/HS diet could be more susceptible to DSS-induced colitis in order to evaluate impact of nutrition on sensitivity of mice to gut inflammation. DSS administration (1% in drinking water for 9 days) in WT and CEABAC10 mice receiving HF/HS diet was found to be associated with significant clinical changes, including a significantly greater body weight loss, presence of diarrhea, and appearance of fecal blood compared with mice fed a conventional diet (Fig. 4A). The Disease Activity Index (DAI) score of WT (10.63 ± 0.65) and CEABAC10 (11.5 ± 0.5) mice fed an HF/HS diet was significantly higher compared to WT (4.0 ± 0.85) and CEABAC10 (8.4 ± 0.6) mice fed a conventional diet (Fig. 4B). Moreover, pro-inflammatory cytokines IL-6, KC and IL-1β were present in higher amounts in colonic mucosa of DSS- HF/HS treated mice (Fig. 4C). Taken together, these findings indicate that a Western diet led to an exacerbation of gut inflammation following chemically-induced colitis.

### Western diet decreases production of SCFA by intestinal microbiota and alters Treg population in mesenteric lymph nodes (MLN)

**Figure 5:**
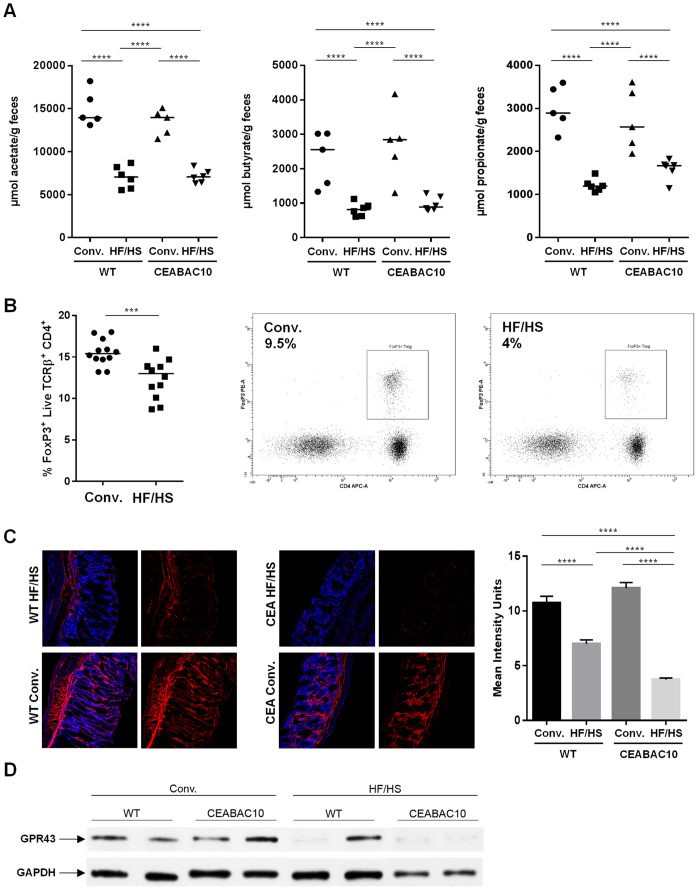
Western diet decreases the level of SCFA produced by intestinal microbiota modulating immune response. (**A**) Acetate, Butyrate and Propionate in fecal samples were measured by gas chromatography, (**B**) Treg population in MLN of mice was analyzed by flow cytometry and representative plots gated were obtained on live TCRβ^+^ MLN cells from conventional or HF/HS fed mice, (**C**) GPR43 receptor expression was visualized by confocal microscopy and (**D**) image intensity was quantified using the ImageJ software. *P < 0.05; **P < 0.01; ***P < 0.001, ****P < 0.0001.

Quantification of SCFA in fecal samples by gas chromatography showed significant decrease in concentrations of the 3 main SCFA from WT and CEABAC10 mice under a HF/HS diet (N = 6) compared with WT and CEABAC10 mice fed a conventional diet (N = 5) (2-fold (WT) and 1.9-fold (CEABAC10) decrease for acetate; 2.5-fold (WT) and 1.7-fold (CEABAC10) decrease for propionate; 2.8-fold decrease (WT and CEABAC10) for butyrate) (Fig. 5A). Of note, no significant differences in concentrations of other minor SCFA were observed (iButyrate, Valerate, iValerate, Caproate, iCaproate, Heptanoate, Table 1). This was paralleled with decreased in Treg population in MLN of mice fed an HF/HS diet (*P* = 0.0008) (Fig. 5B). Because SCFA activate cells *via* GPR such as GPR43 and to better understand the role of SCFA in intestinal inflammation, we next quantified the GPR43 receptor expression of colonic mucosa tissues^19^.

### Expression of SCFA receptor GPR43 is decreased in mice fed a Western diet as well as CD patients

**Figure 6:**
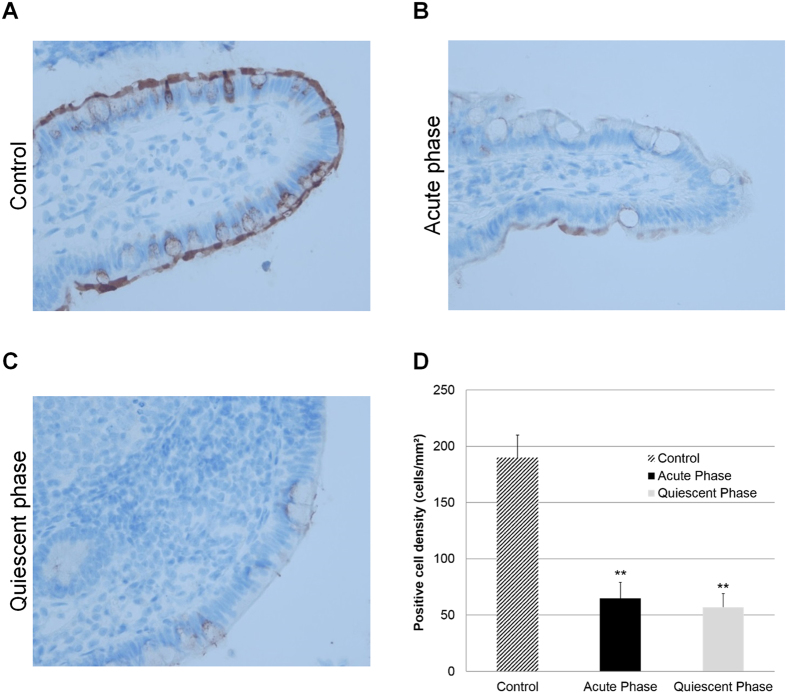
GPR43 expression in the intestinal biopsies of patients with CD and controls. (**A**) GPR43 immunohistochemical staining of TMA from ileum biopsies of controls, (**B**) patients in the acute inflamed phase and (**C**) patients in the quiescent phase of CD. Each spot shows representative tissue immunostaining for GPR43 and for secondary antibody alone (**A–C**, immunoperoxidase, original magnification ×400). Quantification of GRP43 immunostaining using the Spot Browser software in TMA from ileum biopsies of controls and patients in the acute or quiescent phase of CD, **P < 0.001.

GPR43 receptor expression was globally reduced in mice treated with HF/HS diet (N = 6) compared to mice fed a conventional diet (N = 5) (1.5-fold decrease in WT mice and 3.2-fold decrease in CEABAC10 mice; *P* < 0.0001). This was quantified on confocal images by assessing global fluorescence intensities (Fig. 5C) and confirmed by western blot analysis (Fig. 5D). Thereby, we studied GPR43 expression in human intestinal biopsies. GPR43 immunostaining on ileal mucosa were processed and immunohistochemistry showed strong staining of GPR43 in ileal biopsies from control patients, primarily at the epithelium level (Fig. 6A). Strong epithelial staining was observed in all of 41 control patients. In contrast, ileal biopsies taken from active CD (Fig. 6B) and in quiescent CD (Fig. 6C) showed weaker GPR43 expression. In patients with active CD, only a weak GPR43 immunostaining was observed in 66/72 (92%) individuals, whereas in patients with quiescent CD, a weak GPR43 immunostaining was observed in 52/61 (85%) individuals. Concerning positive cell densities in ileal biopsies, quantification of GPR43 immunostaining indicated that numbers of GPR43-positive cells in patients in acute or quiescent phase of CD were similar and significantly lower than those observed in controls (Fig. 6D).

### Pharmacological GPR43 activation prevents gut inflammation

**Figure 7:**
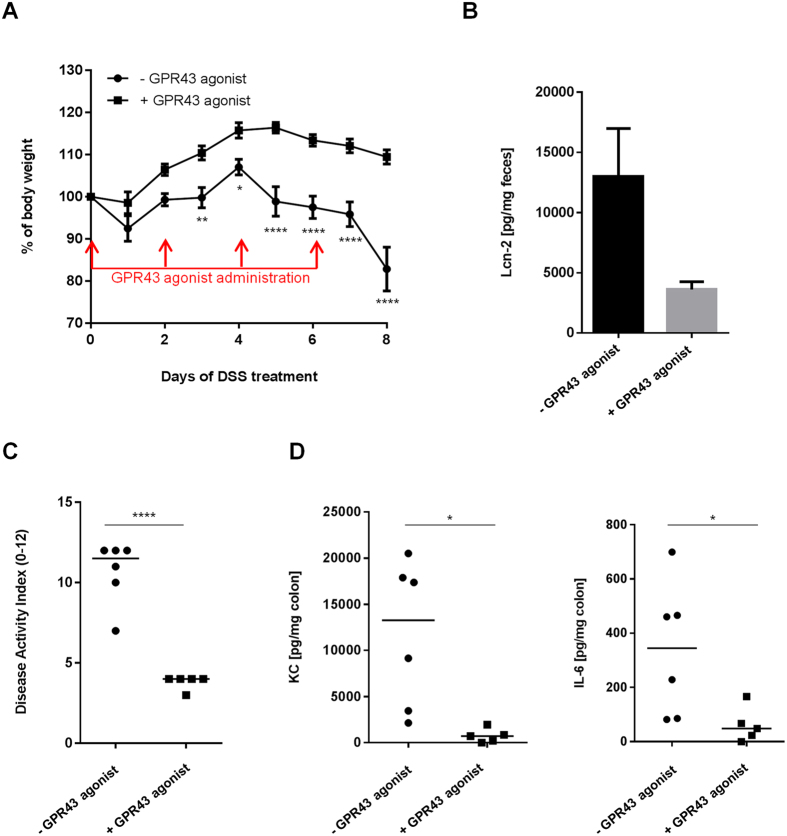
Pharmacological GPR43 activation prevents gut inflammation. (**A**) Evolution of body weight, (**B**) Fecal Lcn-2 levels, (**C**) DAI at day 8 after the beginning of the treatment and (**D**) IL-6 and KC cytokine release by colonic mucosa. *P < 0.05; **P < 0.01; ***P < 0.001, ****P < 0.0001.

To further investigate potential protective role of SCFA and GPR43 receptor in context of inflammation, mice were treated with or without an agonist of GPR43. WT mice treated with the GPR43 agonist (N = 5) and fed a conventional diet were less susceptible to DSS-induced colitis than mice receiving the vehicle only (N = 6). Indeed, signs of colitis were significantly decreased following GPR43 agonist administration. Body weight loss was higher in mice that did not receive the GPR43 agonist (Fig. 7A). The Lcn-2 level in fecal samples and the DAI score were 3.5-fold reduced in mice treated with the GPR43 agonist compared with mice receiving the vehicle only (*P* = 0.0635). Amounts of pro-inflammatory KC and IL-6 cytokines released by colonic mucosa were decreased in mice receiving the agonist (Fig. 7B–D). These results indicate that GPR43 receptor activation by microbiota-produced SCFA is crucial to protect the gut against intestinal inflammation.

## Discussion

The mechanism by which the western lifestyle may play a role in the dramatically increasing prevalence of CD is poorly understood, but it deserves special attention to identify new therapeutic targets that can be used clinically. Commensal bacteria and the immune system have co-evolved and established a symbiotic relationship, but this tenuous equilibrium can be disturbed by environmental factors associated with a Western life style^20^.

Many studies, including our own, have shown that the ileal mucosa of a subgroup of CD patients was abnormally colonized by *E. coli* strains that possess adhesive and invasive properties^8^,^11^,^21^,^22^,^23^. Among the factors associated with a Western lifestyle, diet plays a dominant role in influencing the composition of the gut microbiota, as previously reported in a comparative microbiota study of five inbred mouse strains that were deficient for genes relevant to host-microbial interactions (MyD88^−/−^, NOD2^−/−^, ob/ob and Rag1^−/−^)^24^. Consumption of a HF/HS diet altered the gut microbiota despite differences according to host genotype^24^. This is consistent with our observations, which showed variations in the overall composition of intestinal microbiota associated with the mucosa in both WT and CEABAC10 mice following a HF/HS diet for an 18 weeks period. We also observed that this diet results in a significant increase in the *E. coli* populations colonizing the intestinal mucosa, particularly in CEABAC10 mice. Abnormal expression of *CEACAM6* is required to select overgrowth of mucosa-associated *E. coli*, reinforcing the relevance of the use of transgenic mice expressing human CEACAM6 (CEABAC10) in the context of host susceptibility to *E. coli* colonization. Taken together, this finding suggests that the emergence of AIEC pathobiont bacteria in a predisposed host overexpressing CEACAM6 could be partly selected by the eating habits of the past 50 years in industrialized countries. Of note, the HF/HS diet is characterized both by elevated fat and sugar intake but also by a decreased amount of dietary fiber, we thus reasoned that this last point may lead to a decrease in the production of SCFA, bacterial fermentation products derived from anaerobic bacteria fermenting dietary fibers. However, HF/HS diet used in the study of Parks *et al.* had the same fiber content than control diet and lead to modification of gut microbiota composition and global gene expression^25^. It has been reported that animal models of diet-induced obesity and metabolic disorders have shown that HF/HS diet dramatically changes the gut microbiota composition^26^. HF/HS diet induces also dysbiosis in mucosa-associated microbiota, associated with a thinner and less protective mucus layer and has a crucial role in increased permeability, which results in low-grade inflammation and metabolic disorders^18^,^27^,^28^. Taken together, we suggest that depletion of fiber could partially explain the decrease of SCFA concentrations, but cannot explain the impact of HF/HS diet on global gut dysfunction by itself.

There have been mixed reports regarding the roles of SCFA in the regulation of gut inflammation, possibly due to the distinct effects of these molecules according to their concentration. Indeed, butyrate administration can increase IL-1β and IL-6 expression in the gut via the parenteral route^29^, whereas others studies have reported an anti-inflammatory property of butyrate on human monocytes^30^. SCFA have been reported to have anti-inflammatory properties with an attenuation of production of inflammatory cytokines TNF-α, IL-6 and IFN-γ^31^. Here, we show that a HF/HS diet decreases the acetate, butyrate and propionate concentrations in the mouse gastrointestinal tract. Previous reports have already linked SCFA production to diet and/or diet-mediated changes in the composition of gut microbiota^32^,^33^. Recently, it has also been demonstrated that SCFA can regulate the size and function of colonic Treg populations and protect against colitis in a SCFA receptor-dependent manner in mice^34^. In mice, the butyrate and propionate produced by commensal microorganisms during starch fermentation are able to potentiate extrathymic differentiation of Treg cells^35^. Treg cells expressing the transcription factor FoxP3 have a key role in limiting inflammatory responses in the intestine. In the present study, the impact of HF/HS diet on SCFA concentrations was associated with a decrease FoxP3^+^ Treg population in mesenteric lymph nodes of mice under a HF/HS diet. The combined effects of SCFA are thought to create an overall tolerogenic gut environment by modulating the barrier function, T cell immunity, and neutrophil recruitment, thus helping to prevent infection by pathogens and/or invasion by commensal bacteria. By impacting SCFA production, a HF/HS diet and more generally a western lifestyle could deregulate inflammation in gut mucosa.

SCFA can directly enter within eukaryotic cells by diffusion, but they can also activate cells through cell-surface receptors. G-protein-coupled receptors such as GPR41 and GPR43 have been described to be activated by SCFA^36^ and several studies have demonstrated an anti-inflammatory role for the GPR43 receptor^19^,^37^. The GPR43 binding of SCFA potentially provides a molecular link among the diet, gastrointestinal bacterial metabolism, and immune and inflammatory responses. In this study, we have demonstrated a decreased expression of GPR43 both in mice that were fed a HF/HS diet and in patients with CD. Under this specific diet, the mice exhibit a heightened sensitivity to DSS-induced colitis, and their microbiota, once transferred in axenic mice, increases susceptibility to AIEC LF82 colonization as previously described in CEABAC10 model^18^. These results are consistent with the literature, which reports that GPR43 deficient mice are much more sensitive to DSS-induced colitis than wild-type mice are^19^. In addition, GPR43 deficient mice are not able to efficiently eliminate *Citrobacter rodentium* following bacterial infection^38^. In fact, GPR43 deficient mice failed to induce an acute inflammatory response to clear the pathogens at early time points. This would lead to the delayed clearance and increased invasion of the pathogen into tissues. In parallel, we can suggest that the decreased in GPR43 expression linked to a Western diet, combined with the overgrowth of *E. coli*, could explain the abnormal colonization of the ileal mucosa in CD patients by AIEC strains. Moreover, in other contexts of chronic diseases such as allergies, it has been shown that mice fed a high-fiber diet had increased circulating levels of SCFA and were protected against allergic inflammation in the lung, whereas a low-fiber diet decreased the SCFA levels and increased allergic airway disease^39^. Our findings demonstrate that a Western diet leads to decrease protective SCFA producing bacteria and could promote inflammatory responses in the gut.

GPR43 is involved in disease-relevant physiological processes such as lipolysis, adipogenesis, hormone secretion, and inflammation. GPR43 agonists have potential as therapeutics for the treatment of type II diabetes and obesity^40^. It remains to be elucidated whether this receptor is valuable as a drug target. In addition, drug discovery research on SCFA receptors is unclear on the direction of modulation - agonism vs. antagonism – that would yield therapeutic benefits. In the present study, we showed that pharmacological treatment with an agonist of the GPR43 receptor partially protects mice from the intestinal inflammation induced by DSS treatment. These data are consistent with the literature, which showed that the stimulation of GPR43 by SCFA was necessary for the normal resolution of certain inflammatory responses because GPR43-deficient mice showed exacerbated or unresolved inflammation in models of colitis, arthritis and asthma^19^. This finding seemed to be related to the increased production of inflammatory mediators by GPR43 deficient immune cells, and increased immune cell recruitment. Germ-free mice, which are devoid of bacteria and expressed little or no SCFA, showed a similar dysregulation of certain inflammatory responses. The GPR43 binding of SCFA potentially provides a molecular link among diet, gastrointestinal bacterial metabolism, and immune and inflammatory responses. GPR43 has been implicated in the regulation of fatty-acid and glucose homeostasis in adipose tissue and the intestine and may thus have potential therapeutic relevance in the treatment of type 2 diabetes, insulin resistance and obesity. A similar therapeutic strategy should be evaluated in the context of IBD. Indeed, activation of the GPR43 receptor pathway could be used as a new strategy to treat CD patients colonized by AIEC.

## Methods

### Mice, treatments and ethics statement

Five-week-old C57BL/6 female mice were purchased from Charles River Laboratories for reproduction with heterozygote CEABAC10 transgenic males. Litter-mates of >10^th^ backcross were used for experimentation. Animals were separated by sex and genotype at 4-5 weeks of age. Five-week-old germ-free (GF) C57BL/6 female mice were provided by breeding facilities of the Anaxem platform of INRA (Micalis Unit, Jouy-en-Josas, France). All mice were maintained under specific pathogen-free conditions in the animal care facility at Université d’Auvergne (Clermont-Ferrand, France). Cages were protected with filters to avoid contamination with fecal pellets.

Purified Diet 230 HF (HS/HS) (Safe Industry, France), was used as Western diet, the nutritional composition of which is (in kcal/kg) 13.1% proteins, 60.6% lipids and 26.3% carbohydrates, primarily saccharose and thus contains high levels of fat and simple sugars with a caloric intake of 5317 kcal/kg and a fiber content of 0.017%. Conventional diet (Conv.) was purchased from Safe (3.9% of fiber). Both diets were given for 18 weeks to 5-week-old mice.

### Induction of chemical colitis and clinical assessment of colonic damages

Dextran Sodium Sulfate (DSS: molecular mass = 36,000–50,000 daltons, MP Biomedicals) is frequently used as a model to study inflammatory bowel diseases. Colitis was induced in mice by DSS administration at 1 or 2% in the drinking water for 9 days, depending on the experiment. The body weight and clinical symptoms of colitis (diarrhea and bloody stool) were monitored during the course of the treatment. When the mice attained 80% of their initial weight or at the end of DSS treatment, they were euthanized by cervical dislocation. The Disease Activity Index (DAI) was established to evaluate colonic damages by combining the scores of body weight loss, stool consistency and stool blood. Rectal bleeding was assessed by the Hemocult II test (SKD SARL). This clinical activity score ranges from 0 (healthy) to 12 (greatest activity of colitis) (Table 2).

### GPR43 agonist administration in mice

Mice were orally challenged, every two days for one week, with 5 mg/kg/day of GPR43 agonist (Calbiochem, Millipore) dissolved in 50% of dimethyl sulfoxide (DMSO) or with DMSO only. The body weight of mice was followed throughout the protocol. After this treatment, mice were euthanized by cervical dislocation.

### Fecal transplantation, infection and colonization evaluation of Germ-free mice

Ten germ-free (GF) adult female C57BL/6 mice, aged 8 weeks, without impaired functions were kept in germ-free isolators with controlled light, temperature and humidity. The ten mice were randomly separated into 2 groups of 5 individuals. Each GF mice was then inoculated by a single intragastric gavage with 300 μL of 100-fold diluted fecal sample from mice fed with a conventional or with the HF/HS diet (100-fold dilution of fecal sample in anaerobic mineral solution, approximately 10^9^ CFU/mL). Mice were bred for three weeks in sterile isolators and were fed irradiated standard diet for germ-free rodents (SAFE, Augy, France) with free access to sterilized water.

Three weeks after inoculation, the establishment of the gut microbiota in mice was controlled by enumerating the total anaerobes in fecal samples in liquid medium using anaerobic techniques (100% O_2_-free CO_2_ gas)^41^. Total anaerobes were enumerated by the Most Probable Number estimation with three tubes inoculated per dilution^42^. The total number of anaerobic bacteria established in mice ranged from to 2.10^10^ to 1.25.10^11^/g of feces.

At age of 11 weeks, transplanted mice were orally challenged during three consecutive days with 10^9^ ampicillin-erythromycin-resistant AIEC LF82 strain isolated from a CD patient. Five days after this cycle step of infection, mice were infected for a second cycle, consisting of three consecutive days with 10^9^ AIEC LF82. The weight of the mice was monitored throughout the treatment. Bacterial persistence was evaluated at 3 days post-infection in the fecal samples. LF82 bacteria were enumerated by plating serial dilutions on LB agar medium containing 100 μg/ml ampicillin and 20 μg/ml erythromycin and were incubated overnight at 37 °C. The number of bacteria was determined by counting the colony-forming units (CFU).

### Patients and immunohistochemistry on TMA

All included patients (71 men and 62 women) were hospitalized in the Gastroenterology Department (Archet II Hospital, University of Nice Sophia Antipolis, France) and provided signed agreement for this study. The protocol was approved by the Ethics Committee of the University of Nice Sophia Antipolis. The methods were carried out in accordance with the approved guidelines. Intestinal biopsies were obtained from macroscopically terminal ileum inflamed mucosa of 72 patients with active CD and from macroscopically terminal ileum non-inflamed mucosa of 61 patients with quiescent CD. Mean age of patients was 40 years (range 20–62) and mean disease duration was 11 years (range 2–23). Patients were all French Caucasians. Biopsies were taken from ileum of 41 control patients that had no significant pathological findings following an endoscopic examination for changes in stool habits, abdominal pain, upper gastrointestinal bleeding or cancer surveillance. Expression of GPR43 was quantified by immunohistochemistry on TMA.

### Immunohistochemistry on TMA

Representative intestinal biopsies obtained for each individual in building TMA were selected from hematoxylin and eosin stained sections. Briefly, one tissue core (600 μm in diameter) was obtained from each specimen from the upper part of the mucosa; pits and glands were always cut longitudinally. The TMA were built as previously described. Immunohistochemical (IHC) methods were performed on serial 4 μm deparaffinized TMA sections processed as described. Anti-GPR43 (Santa Cruz Biotechnology), and secondary antibody alone were used as a negative control. To measure the histological disease activity, the scoring system for histological abnormalities in CD mucosal biopsy specimens was used. Slides were analyzed with an image analysis workstation (Spot Browser version 7; Alphelys, Plaisir, France), as described.

### Quantification of fecal Lcn-2 and cytokine release

Fecal lipocalin-2 (Lcn-2) was measured by ELISA experiments to detect low-grade inflammation during the course of the treatment. Frozen fecal samples were reconstituted in PBS containing 0.1% Tween 20 (100 mg/ml) and disrupted to obtain a homogenous fecal suspension. These samples were then centrifuged for 10 min at 12,000 rpm and 4 °C. Clear supernatants were collected and stored at −80 °C until analysis. Lcn-2 levels were estimated in the supernatants using a mouse Duoset Lcn-2 ELISA kit (R&D Systems, Minneapolis, MN). Fecal samples were diluted in the kit-recommended reagent diluent (1.0% BSA in PBS, pH 7.2–7.4). For pro-inflammatory cytokine quantification (IL-6, KC, IL-1β), weighted colonic samples were placed in Dulbecco’s modified Eagle medium (DMEM) with antibiotics (gentamicin 50 mg/ml and antibiotic cocktail, PAA) and incubated for 24 h at 37 °C. The cytokine levels were quantified in the supernatant using ELISA kits from R&D systems following the manufacturer’s instructions.

### Determination of SCFA concentration

Quantification of SCFA was performed by gas chromatography in supernatants of fecal samples reconstituted in PBS and disrupted for 10 min at 4 °C. Samples were then centrifuged for 5 min at 13,000 g at 4 °C. SCFA production in stool samples was determined by gas chromatography. Samples supplemented with internal standard (2-ethyl butyric acid at 49 mM) were deproteinized by addition of saturated phosphotungstic acid (500 g/L), centrifuged at 9000 g for 20 min and then supernatants were filtered (0.45 μm). Samples were run through a 6890 Series GC System (HP, USA) fitted with a HP-INOWax column (0.25 mm × 30 m × 0.25 μm) and flame ionization detector. Helium was used as carrier gas and was delivered at a flow rate of 2 mL/min. Injector and detector were set at 250 °C. Column was maintained in an oven with a temperature gradient ranging from 110 to 240 °C. One micro liter quantity of each sample was injected with a run time of 14.3 min. Peaks were integrated using HP ChemStation software. Fatty acids were quantified by comparing their peak areas with the corresponding standards.

### Analysis of GPR43 and *E. coli* colonization by confocal microscopy

Colonic and ileal 5 μm frozen sections of mice were stained using rabbit anti-human GPR43 polyclonal antibody (Santa Cruz Biotechnology), goat anti-*E. coli* polyclonal antibody (AbD Serotec) and Cy3-conjugated anti-rabbit IgG (Vector Laboratories) and A488-conjugated anti-goat IgG (Molecular Probes) as secondary antibodies, respectively. Tissues were observed with confocal microscope (LEICA SPE) and signal quantification was performed using ImageJ software.

### Microbiota composition analyses by Illumina Sequencing

Proximal colon tissues were washed with PBS and used for microbiota analysis. NucleoSpin® Tissue (Macherey-Nagel GmbH & Co.) was used to extract and purify genomic DNA following manufacturer’s instructions. An overnight incubation with lysis buffer (in this condition, the tissue is completely solubilized) was performed to optimize bacterial DNA extraction and lysis temperature was increased to 95 °C for Gram-positive bacteria, which are difficult to lyse. DNA samples were characterized by profiling microbial community based on of 16S ribosomal RNA gene by using Illumina MiSeq sequencing. The 16S rRNA gene V4 variable region PCR primers 515/806 (OR OTHER PRIMER SELECTED) with barcode on the forward primer were used in a 30 cycle PCR using the HotStartTaq Plus Master Mix Kit (Qiagen, USA) under the following conditions: 94 °C for 3 minutes, followed by 28 cycles of 94 °C for 30 seconds, 53 °C for 40 seconds and 72 °C for 1 minute, after which a final elongation step at 72 °C for 5 minutes was performed. After amplification, PCR products were checked in 2% agarose gel to determine the success of amplification and the relative intensity of bands. Multiple samples were pooled together (e.g., 100 samples) in equal proportions based on their molecular weight and DNA concentrations. Pooled samples were purified using calibrated Ampure XP beads. Then the pooled and purified PCR product was used to prepare DNA library by following Illumina TruSeq DNA library preparation protocol. Sequencing was performed at MR DNA (www.mrdnalab.com, Shallowater, TX, USA) on a MiSeq following the manufacturer’s guidelines. Sequence data were processed using MR DNA analysis pipeline (MR DNA, Shallowater, TX, USA). In summary, sequences were joined, depleted of barcodes then sequences < 150bp removed, sequences with ambiguous base calls removed. Sequences were denoised, OTUs generated and chimeras removed. Operational taxonomic units (OTUs) were defined by clustering at 3% divergence (97% similarity). Final OTUs were taxonomically classified using BLASTn against a curated database derived from GreenGenes, RDPII and NCBI (www.ncbi.nlm.nih.gov, DeSantis *et al.* 2006, http://rdp.cme.msu.edu).

The software package, Quantitative Insights Into Microbial Ecology (QIIME) was used for filtering and analysis of attained sequences. Alpha diversity rarefaction curves were produced by plotting several diversity metrics against the number of sequences considered from a sample. Subsequent analyses of diversity were performed at a depth of 20,000 sequences per sample. Alpha diversity was studied using the phylogenetic diversity measurement, PD_whole_tree, and by the number of observed species and the species richness estimator Chao1. Beta diversity was performed with QIIME using a Principal Coordinate Analysis (PCoA), measuring dissimilarities at phylogenetic distances based on unweighted UniFrac analysis. QIIME was used also to provide the relative abundance of bacterial groups at different taxonomic levels between each group of mice according to diets and genotypes.

### Treg cells analysis

Mesenteric lymph nodes (MLN) were harvested and crushed in ice-cold PBS through nylon-meshed cell strainers. MLN-derived cells were washed in PBS and stained with fixable Viability Dye eFluor450 (eBioscience) according to manufacturer’s instructions. Cells were then washed, permeabilized and incubated at 4 °C with anti-CD16/CD32 before staining with anti-TCRβ APC-eFluor780, anti-CD4 APC and anti-FoxP3 PE (all antibodies from eBioscience). Data were acquired on a BD LSR II flow cytometer, and FACS Diva software (Becton-Dickinson) was used for analysis.

### Statistical Analysis

Statistical analyses were performed using GraphPad Prism 6.00 (GraphPad Software, San Diego, CA, USA) software package for PC. Values are expressed as mean ± SEM or median. Data were compared using Student’s t-test analysis or non-parametric one-way analysis of variance Mann-Whitney test when appropriate. ANOVA was used for intergroup comparison. Differences observed in GPR43 immunostaining, assays were compared using Student’s t-test. Values are expressed as the mean ± SEM of ‘n’ number of experiments. Association of GPR43 expression with categorical pathological features was made using χ^2^ analysis. Calculations and analyses were performed with SPSS 11.5 for Windows, and, when appropriate, were two-tailed. Student’s t-test was used to analyze statistical significance between invasion levels. P values ≤0.05 were considered statistically significant.

### Ethical considerations

Informed consent was obtained from all subjects before conducting the experiments. TMA protocol was approved by the local ethic committee of the University of Nice Sophia Antipolis, France. Animal protocols were carried out in strict accordance with the recommendations of the Guide for the Care and Use of Laboratory Animals of the University of Clermont-Ferrand, France and were approved by the Committee for Research and Ethical Issues of the Department of Auvergne (CEMEA Auvergne) following international directive 86/609/CEE (n°CE16-09).

## Additional Information

**How to cite this article**: Agus, A. *et al.* Western diet induces a shift in microbiota composition enhancing susceptibility to Adherent-Invasive *E. coli* infection and intestinal inflammation. *Sci. Rep.* **6**, 19032; doi: 10.1038/srep19032 (2016).

## Supplementary Material

Supplementary Information

## Figures and Tables

**Table 1:** Short-chain fatty acids concentrations.

SCFA (μmol/g feces ± SD)	WT Conv.	WT HF/HS	CEA Conv.	CEA HF/HS
Acetate	15033 ± 2095	7046 ± 1285	13434 ± 1517	7143 ± 769
Propionate	3006 ± 518	1216 ± 150	2738 ± 720	1593 ± 235
Butyrate	2300 ± 799	818 ± 195	2707 ± 1036	978 ± 208
iButyrate	123 ± 23	239 ± 290	103 ± 24	159 ± 41
Valerate	243 ± 54	151 ± 86	176 ± 45	181 ± 64
iValerate	119 ± 30	124 ± 15	107 ± 36	170 ± 32
Caproate	90 ± 13	171 ± 30	86 ± 23	191 ± 41
iCaproate	0	0	0	0
Heptanoate	0	0	34 ± 57	76 ± 136

**Table 2:** Disease Activity Index (DAI) scoring.

Symptom/score	Characteristics
Body weight loss	
0	No loss
1	1–5% loss of body weight
2	5–10% loss of body weight
3	10–20% loss of body weight
4	> 20% loss of body weight
Stool consistency	
0	Normal feces
1	Loose stool
2	Watery diarrhea
3	Slimy diarrhea, little blood
4	Severe watery diarrhea with blood
Blood in stool	
0	No blood
2	Presence of blood assessed by Hemoccult II test
4	Visible bleeding
