# Supplementary material for: Western diet induces a shift in microbiota composition enhancing susceptibility to Adherent-Invasive E. coli infection and intestinal inflammation
Source: Sci Rep. 2016 Jan 8;6:19032. doi: 10.1038/srep19032 (PMC4705701; doi:10.1038/srep19032)
Supplement: Supplementary Information [file srep19032-s1.pdf]

Supplementary Information

Western diet induces a shift in microbiota composition enhancing susceptibility to Adherent-Invasive *E. coli* infection and intestinal inflammation.

Allison Agus[1], Jérémy Denizot[1], Jonathan Thévenot[1,2], Margarita Martinez-Medina[1], Sébastien Massier[1], Pierre Sauvanet[1,3], Annick Bernalier-Donadille[4], Sylvain Denis[2], Paul Hofman[5], Richard Bonnet[1,6], Elisabeth Billard[1,7], Nicolas Barnich[1,7*]

[1]Clermont Université, M2iSH, UMR 1071 INSERM/Université d'Auvergne, Clermont-Ferrand, France Unité Sous Contrat 2018 Institut National de la Recherche Agronomique, Clermont-Ferrand, France; [2]Clermont Université, Université d'Auvergne, Centre de Recherche en Nutrition Humaine Auvergne, EA 4678 CIDAM, Conception Ingénierie et Développement de l'Aliment et du Médicament, Clermont-Ferrand, France ; [3]Digestive Surgery Department, Centre Hospitalier Universitaire, Clermont-Ferrand 63000, France ; [4]UR454 Microbiology Division, INRA, Research Centre of Clermont-Ferrand-Theix, 63122 Saint Genès-Champanelle, France ; [5]Laboratory of Clinical and Experimental Pathology and Hospital-Related Biobank (BB 0033-00025), Pasteur Hospital, and IRCAN CNRS UMR 7284, Inserm U1081, Nice Sophia Antipolis University, France ; [6]Bacteriology Department, Centre Hospitalier Universitaire, Clermont-Ferrand 63000, France, [7]Institut Universitaire de Technologie, Génie Biologique, Aubière, France.

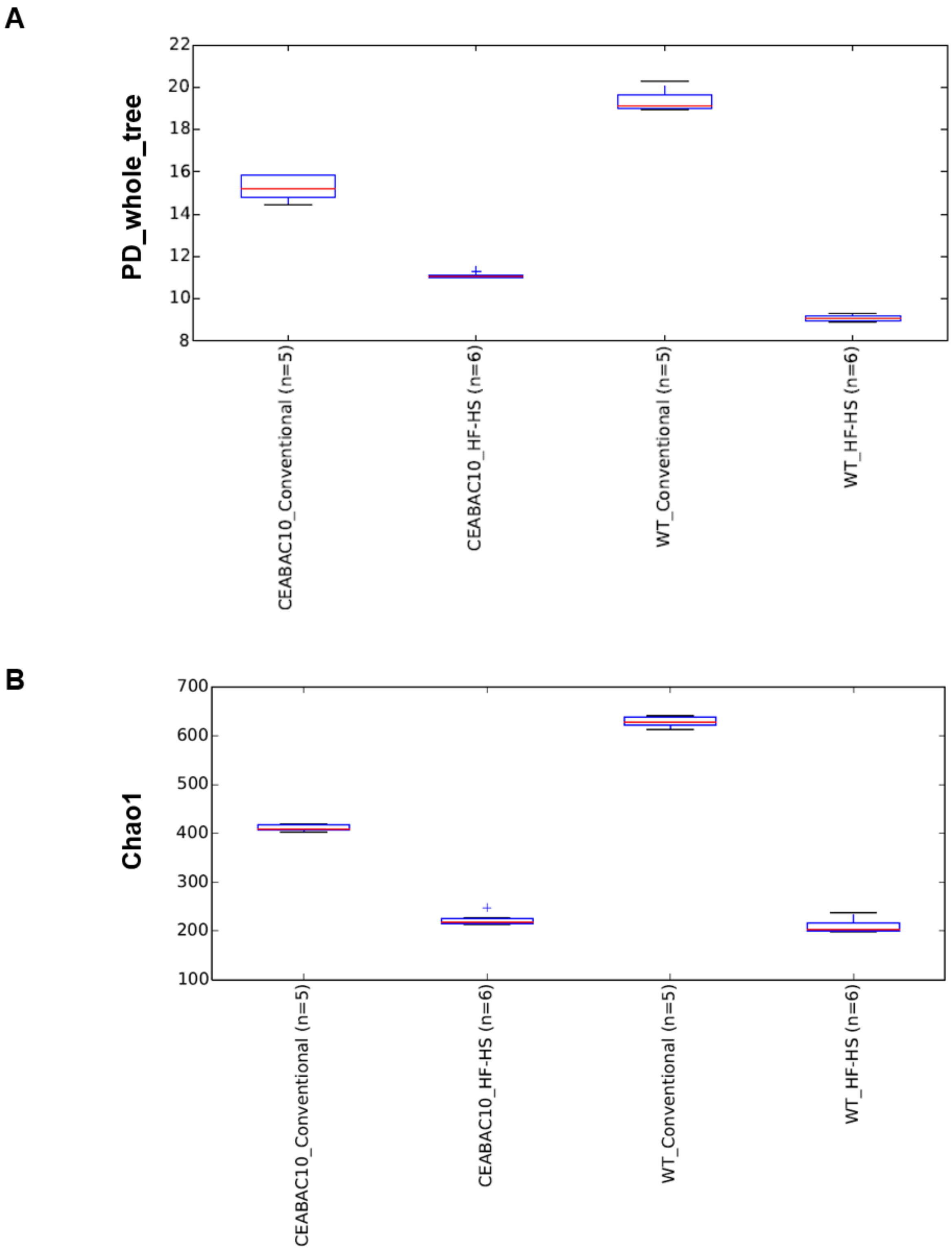

Figure S1: (A) Microbiota alpha diversity measured by the phylogenetic distance and (B) the expected richness.
